# Does Postponement of First Pregnancy Increase Gender Differences in Sickness Absence? A Register Based Analysis of Norwegian Employees in 1993–2007

**DOI:** 10.1371/journal.pone.0093006

**Published:** 2014-03-25

**Authors:** Anja M. S. Ariansen, Arnstein Mykletun

**Affiliations:** 1 Department of Sociology, University of Bergen, Bergen, Norway; 2 Norwegian Institute of Public Health (FHI), Bergen, Norway; 3 University of New South Wales, School of Psychiatry, Sydney, Australia; Iran University of Medical Sciences, Iran (Islamic Republic Of Tehran)

## Abstract

**Background:**

From 1970–2012, the average age at first delivery increased from 23.2–28.5 in Norway. Postponement of first pregnancy increases risks of medical complications both during and after pregnancy. Sickness absence during pregnancy has over the last two decades increased considerably more than in non-pregnant women. The aim of this paper is twofold: Firstly to investigate if postponement of pregnancy is related to increased sickness absence and thus contributing to the increased gender difference in sickness absence; and secondly, to estimate how much of the increased gender difference in sickness absence that can be accounted for by increased sickness absence amongst pregnant women.

**Methods:**

We employed registry-data to analyse sickness absence among all Norwegian employees with income equivalent to full-time work in the period 1993–2007.

**Results:**

After control for age, education, and income, pregnant women's sickness absence (age 20–44) increased on average 0.94 percentage points each year, compared to 0.29 in non-pregnant women and 0.14 in men. In pregnant women aged 20–24, sickness absence during pregnancy increased by 0.96 percent points per calendar year, compared to 0.60 in age-group 30–34. Sickness absence during pregnancy accounted for 25% of the increased gender gap in sickness absence, accounting for changes in education, income and age.

**Conclusions:**

Postponement of first pregnancy does not explain the increase in pregnant women's sickness absence during the period 1993–2007 as both the highest level and increase in sickness absence is seen in the younger women. Reasons are poorly understood, but still important as it accounts for 25% of the increased gender gap in sickness absence.

## Introduction

Norway's high levels of fertility and female employment is often cited in support of the success story of the Nordic model [1]. The employment rate has recently reached 73% among women and 77% among men [2], and in 2008 the fertility rate was 1.96 [3]. However, high and growing levels of sickness absence is also part of this picture, and entails public costs [4]. To what extent increased sickness absence over the last two decades primarily applied to women in general is currently being debated [5], [6], while the recent increase in sickness absence among pregnant women is less controversial [7], [8]. In order to facilitate fertility as well as female employment, it is imperative to better understand causes of the increase in pregnancy related sickness absence, and also causes for sickness absence in general.

Norway is a social democratic welfare state [9], which provides generous health care services and pensions to reduce social inequality [10]. Accordingly, sick listed employees get their wage fully compensated for a year, and high and growing levels of sickness absence entail substantial public expenses.

Previous research on pregnant women's sickness absence has investigated the impact of economic incentives [11]–[14], while the possible impact of higher age for pregnant women's increased absence has received less attention. The impact of higher age on pregnant women's sickness absence is only partly investigated, and the impact of pregnancy on the increasing gender differences is not examined. Moreover, increasing age among pregnant women applies to most western countries, not only Norway [15], [16].

Higher age among pregnant women follows from the educational expansion, which recently has occurred in European and North-American countries. Women increasingly take part in higher education or career start prior to first pregnancy, thus giving birth later in life, compared to women in previous generations [15]–[17]. As the risk for adverse outcomes tends to intensify with increasing age [18], [19], delayed childbearing might entail growing levels of sickness absence among highly educated pregnant women.

Rieck et.al. [7] and Markussen et.al. [8] have recently found the increase in sickness absence during pregnancy to be strongest in younger women, but the level of sickness absence across age groups of pregnant women was not the focus of attention in their analyses. The strong demographic tendency of postponement of pregnancies implies that a relatively large proportion of more women now give birth at an age with a relatively higher risk of medical complications.

Pregnant women's increased sickness absence in Norway coincides with a discussion about to what extent gender differences in sickness absence are currently increasing and reasons for this eventual increase [5], [6]. The topic is also high on the political agenda, because gender equality largely is a shared political goal in Norway, which lately has resulted in increased levels of education, employment, and income among women [20], and men doing more household work [21].

As sickness absence might have negative side effects in terms of reduced income prospects, social exclusion and reduced career opportunities [22]–[24], women's higher rate of sickness absence represents an obstacle to gender equality in the labor market.

Thus several attempts have been made to explain the gender differences in sickness absence, both in Norway and elsewhere. Contributions in this regard broadly focus on explaining women's heightened sickness absence in terms of one or more of the 5 following approaches: 1) health differences, 2) pregnancy, 3) the double burden of work and family, 4) gender segregation in the labour market and 5) normative threshold for sickness absence.

Analyses from US suggest that biological differences accounts for some of the gender differences in sickness absence [25]. Several studies indicate that biological factors entail health differences between the sexes [26]–[28]. In spite of this, gender differences in sickness absence in Norway is so far not explained by health differences [29]. It is difficult to imagine biological changes accounting for the increased gender difference in sickness absence over the last 3 decades, as the biological differences between the sexes remains rather constant over such a short time period.

The impact of pregnancy on the gender differences in sickness absence is not previously assessed, but the impact is shown to be substantial in Sweden [30], [31]. Parenthood only has a limited impact on Norwegian women's sickness absence [32], [33].

Although the Norwegian labour market is highly segregated, this does not account for women's higher sickness absence [34]. However, occupational changes partly explains the why gender differences have increased, especially the increase that occurred during the 80ies [6]. A majority of women at the workplace seems to entail increased sickness absence, which possibly reflects gender specific attitudes to sickness absence [35], [36]. However, this does not explain much of the gender differences in Norway [36].

In spite of several efforts to solve the puzzle, gender differences in sickness absence in Norway remains largely unexplained. The impact of pregnancy is however not yet assessed in Norway, although pregnancy has had a considerable impact on gender differences in sickness absence in Sweden [30], [31]. The recent increase in pregnant women's sickness absence in Norway [8] further highlights the question of whether the increasing gender differences in sickness absence is mainly due to pregnancy, and whether the growing difference between the sexes is accounted for by the increase among pregnant women.

The first aim of this paper is to examine if pregnant women's increased sickness absence is partly explained by the growing numbers of pregnant women aged 30 and above being more prone to sickness absence. The second aim of the paper is to examine if the increased gender differences in sickness absence is accounted for by increased sickness absence during pregnancy.

## Methods

Our analyses are carried out on the event history data base “FD-Trygd”. This contains information about The Norwegian population, and each individual's job and family characteristics and his/her receipt of welfare benefits. The registrations are carried out by The Norwegian Labour and Welfare Administration, The Norwegian Tax Administration, and Ministry of Health and Care Services in cooperation with the national estimation agency Statistics Norway at the Ministry of Finance.

### Ethics

The Norwegian Data Protection Authority has approved utilisation of the registry data for purposes like this study. Informed consent was not required, because the data were made anonymous. According to Norwegian law, such projects are not subject to approval by the medical ethics committees.

### Data management

The data collection contains detailed information about every sick leave spell covered by the national insurance, as well as information about age, gender, annual salary and educational level, and also date of delivery when this occurred. Due to inadequate information on part-time employment and weekly working hours, part time employees and non-pregnant employees who were on parental leave part of the year were excluded from our analyses. Women were also excluded from the estimates for non-pregnant women the calendar year they became pregnant, as their possible number of sick days in a non-pregnant condition were then reduced. We used an income based inclusion criterion to exclude part-timers.

The full-time income inclusion criterion was based on income from annual salaries only. All employees were only included in the analysis for the years that he or she had exceeded the income inclusion criterion. Sickness absence was estimated for employees only; absence from any additional activities as self-employed was excluded from our analyses. The employees' sickness absence included all records regardless of diagnosis, including episodes where other people's disease was recorded as the cause of the employee's absence. In line with this any days of absence covered by the National Insurance through the pregnancy benefit arrangement were also included. Pregnancy benefit applies to cases where the pregnant woman's working conditions posed a risk to the fetus. Many sick spells (or pregnancy benefit spells) began in one calendar year and ended in the following. In these cases we divided the individual's total number of absence days between the two calendar years, according to the proportions of the spell which occurred prior to and after year end, respectively.

The lower cut-off for full-time income was subject to discussion, as there was no common statuary minimum wage for all employees in Norway during the observation period. Rather, the minimum wage varied with occupational and union membership throughout the period, as unions negotiated different minimum wages in various professions on behalf of their members. Gradually the minimum wage for union members was applied to all workers within specific sectors, in line with The Act on General Application from 1993. The inclusion criterion for our analyses was full-time income above 3.5 Basic Units of the National Insurance, which was adjusted according to the annual increase of employees' salaries each year [37]. The inclusion criterion constituted a gross annual salary of NOK 229 267 before tax in 2007 (about EUR 30 826). This cut-off was about NOK 10000 below the average annual salary of the 10% lowest-paid full-time employed women in 2007, which was estimated by the online calculator of Statistics Norway [38].

Placing the cut-off somewhat below the average salary of these women was decided to ensure inclusion of the vast majority of full-time employed men and women in low-paid occupations in the analyses. Lowering the cut-off further implied including more high-paid part-time employees. However, the limit of 3.5 basic units allowed inclusion also of an unknown, but probably limited, proportion of high-income part-time employees. To ensure that the conclusion did not depend solely on the income inclusion criterion, all analyses were firstly based on the definition of full time employment based on 3.5 basic units cut-off, and then we repeated the main analyses with the higher cut-off of 4 basic units. The change in income limit did not change any of the main findings or conclusions, the most important change of findings being that the proportion of the increased gender gap in sickness absence accounted for by sickness absence during pregnancy increased from 2.5% to 26.6%. In the balance between including too many high-salary part-time employees versus excluding low-income full-time employees, we decided to keep the >3.5 basic unit definition of full-time income, an inclusion criterion for all analyses in this paper. Each individual was only included in the estimates for the years that his or her gross salary exceeded the income cut-off.

### Definitions

In the following analyses births were not registered before 1992. To identify first-time pregnancies we combined household registers from 1992 with birth registers from 1992 and onwards. Only women without children in the household in 1992 and without previous registered births from 1992 onwards were categorised as first-time mothers. If the youngest child registered in the women's household in 1992 were less than 18 years younger than the woman herself we regarded this as a younger sibling rather than offspring. Accordingly the first registered pregnancy of these women from 1992 onwards was also categorised as first-time pregnancy.

The duration of pregnancy obviously varies [39]. In our analyses pregnancy was defined as a period starting from 282 days prior to delivery and until the pregnant women gave birth. This definition equals expected gestational age, which starts the first day of last pregnancy prior to conception and ends the day of delivery, and accordingly extends the average period from conception to birth with 16 days. Defining pregnancy in terms of gestational age suited two purposes in our analyses. Firstly, health professionals in the Norwegian health care system communicating with pregnant women or women who are planning to get pregnant frequently refer to the first day of the last menstrual period before conception as the first day of pregnancy. Thus this measure covers the entire period that many women perceived themselves as undergoing pregnancy, even though it is not medically confirmed yet. To the extent that this perception change their sickness absence, we consider them categorized as pregnant at that time, rather than not. Secondly this definition captures sickness absence among pregnant women who give birth after term as well. In sum, pregnancy possibly influences sickness absence for many women for a period which somehow exceeds the expected period between conception and birth with a few days, and our assessment of pregnancy is suited to capture this. All days of sick leave that occurred from the defined pregnancy start until the woman gave birth or goes on maternity leave was categorised as sickness absence during pregnancy, regardless of diagnosis.

Educational level was categorized in terms of primary school (completed primary school or lower), secondary school (completed secondary school) and higher education (completed bachelor or higher degree). Although job category could be relevant as well, information about occupation is only accessible from 2003 and onwards, and was thus not available for the analyses.

### Statistical analyses

In each of the analyses presented sickness absence was measured as a rate; the number of number of sick days actually covered by the National Insurance insurance the current year is divided on maximum possible sick leave days covered by the national insurance over the same time span. We regarded this estimate as more accurate than estimates based on the individual's contracted working hours as information about the latter is characterized by altered registration practices during the observation period. Each spell was limited to the maximum number of days officially compensated by the national insurance the current year, to avoid outliers due to registration errors.

Descriptive statistics are presented in Tables 1 and 2, while the main findings are presented in graphs. Regression analyses were included in order to estimate regression coefficients, adjust for potential confounding factors, and examine curve-linearity and interactions. As the analyses are based on the entire population and not a sample thereof, it is not relevant to rely on p-values for interpretations related to whether observed trends, associations and interactions are type 1 errors or also present in the general population. However, regression models were applied for purposes of quantification of trends, shapes of associations and interactions. Ordinary least square regression was preferred as the dependent variable of sickness absence is continuous.

**Table 1:** Descriptive statistics of study population.

	1993				2007			
	Men	Non-pregnant women	Pregnant women	Total	Men	Non-pregnant women	Pregnant women	Total
Frequency	481965	262648	25214	769827	507715	320534	31846	860095
Percentage (%)	62.61	34.12	3.28	100	59.03	37.27	3.70	100
Any sickness absence^1^ (% Yes)	10.0	14.2	58.1	13.1	13.4	21.4	67.6	18.5
Sickness absence percentage (%)	1.7	2.4	17.6	2.4	2.9	4.9	25.7	4.5
Age (mean)	33.7	34.4	29.8	33.8	34.1	35.4	31.6	34.5
Earnings (# Basic units^2^)	6.66	5.16	4.85	6.09	7.00	5.48	5.15	6.37
Education (% Higher)	26.58	34.32	41.98	29.73	31.08	49.36	66.23	39.31

1 Both the percentage of employees with any sickness absence at all and the annual sickness absence percentage of sickness absence are based on registrations which are normally counted from day 17 of the spell. More details about the variables are found in the Methods section.

2 One basic unit of income amounted to about EUR 8807 in 2007.

**Table 2:** Percentage points of sickness absence among first-time pregnant women.

	20–24		25–29		30–34		35–39		40–44	
	N	%	N	%	N	%	N	%	N	%
1993	2091	16.73	6393	51.14	3070	24.56	816	6.53	131	1.05
1994	1874	14.69	6473	50.74	3336	26.15	923	7.23	152	1.19
1995	1854	14.49	6377	49.83	3452	26.97	959	7.49	156	1.22
1996	1796	13.8	6476	49.77	3610	27.74	980	7.53	151	1.16
1997	1635	12.57	6321	48.61	3827	29.43	1044	8.03	176	1.35
1998	1528	11.59	6300	47.79	4062	30.81	1129	8.56	164	1.24
1999	1771	12.44	6730	47.27	4388	30.82	1176	8.26	173	1.22
2000	1663	11.65	6661	46.66	4509	31.58	1260	8.83	183	1.28
2001	1582	11.38	6299	45.3	4554	32.75	1292	9.29	177	1.27
2002	1471	10.77	5977	43.77	4758	34.84	1255	9.19	194	1.42
2003	1365	9.82	5869	42.24	5009	36.05	1453	10.46	198	1.43
2004	1261	9.09	5712	41.2	5094	36.74	1562	11.27	236	1.7
2005	1108	8.44	5202	39.61	5058	38.52	1513	11.52	251	1.91
2006	1151	8.57	5356	39.86	4986	37.11	1686	12.55	258	1.92
2007	1259	9.4	5169	38.59	4924	36.76	1713	12.79	330	2.46

According to age group.

The regression analysis presented in Table 3 served to estimate the annual increased in sickness absence among pregnant women, and adjust this estimate for interactions of age and education. In the regression model, year was included as a continuous variable with year as unit. The estimates were also adjusted for income, which is a continuous variable whose value equals the employees earnings measured in number of basic units the current year. One basic unit amounted to about EUR 8 807 in 2007.

**Table 3:** Linear regression model with percentage points of sickness absence as the dependent variable.

	Model 1	Model2	Model 3	Model 4	Model 5	Model 6
Year	0.64		0.94	0.94	0.93	1.70
Age		−0.52	−0.24	−3.93	−3.48	19.28
Income(# Basic units)			−1.39	−1.37	−1.42	−1.42
Secondary school			−7.01	−6.70	−6.18	−882.42
Higher education			−13.47	−12.62	−22.01	−1,203.57
Age x Age				0.06	0.05	0.05
Secondary school x Age					−0.02	37.83
Higher education x Age					0.32	89.84
Year x Age						−0.01
Secondary school x Year						0.44
Higher education x Year						0.59
Secondary school x Year x Age						−0.02
Higher education x Year x Age						−0.04
Constant	−1,261	35.28	−1,839	−1,774	−1,775	−3,308
Observations	188447	188447	188447	188447	188447	188447
R-squared	0.01	0.01	0.07	0.07	0.07	0.08

Only first-time pregnant women included.

Further, education was included in the regression models a set of dummy variables, with primary school as the baseline category, and separate dummies for secondary school and higher education. In the regression analyses, the variable age is a continuous variable, its value equals the current calendar year minus the respondent's year of birth.

Age squared was also included to examine possible curved associations with age. Because the impact of age on sickness absence may differ between pregnant women in different educational groups, the products of age and each of the education dummies were included as interaction terms.

Finally, a three way interaction between calendar year, age and educational level was included to account for the possibility that the interaction between age and educational level vary over time. A three way interaction may be solved by including the two way products of all three variables as separate variables, in addition to the product of all three of them [40]. Thus year by age, age by education, and year by education, as well as the three way interaction term year by age by education was included in the regression model.

The regression analysis presented in Table 4 was conducted to estimate the percentage of the increased gender difference in sickness absence which applied to pregnant women. Here, the dependent variable consisted of average sickness absence percentage and the regression analysis includes pregnant women, non-pregnant women, and men. When estimating the impact of pregnancy on the gender differentials in sickness absence, it was required to take into account that each pregnant woman was working less than a full time equivalent in pregnant condition. Therefore all individuals were weighted in the regression to account not only for their average level of sickness absence, but also the proportion of a working year that they were employed.

**Table 4:** Linear regression model with percentage of sickness absence as the dependent variable.

	Without control variables		With control variables		
	Model 1	Model 2	Model 3	Model 4	Model 5
Year	0.11	0.11	0.14	0.15	0.14
Gender	−264.30	−188.26	−297.04	−290.89	−218.81
Income (# Basic Units)			−0.27	−0.28	−0.27
Secondary school			−1.60	−3.13	−3.03
Higher education			−2.65	−6.06	−7.48
Age			0.03	0.68	0.49
Age x Age				-0.01	−0.01
Secondary school x Age				0.04	0.04
Higher education x Age				0.09	0.13
Gender x Year	0.13	0.09	0.15	0.15	0.11
Pregnancy		−1,087.72			−1,185.80
Pregnancy x Year		0.55			0.61
Pregnancy x Age					−0.40
Constant	−225.32	−225.32	−282.37	−295.71	−291.92
Observations	11452043	11452043	11452043	11452043	11452043
R-squared	0.01	0.06	0.03	0.03	0.08

The coefficients of Woman x Year indicates the annual increase in gender differences in sickness absence before and after control for the annual increase in sickness absence among Pregnant women. With and without control for education, income, age squared, and interactions between education and age, and pregnancy and age.

The coding of the variables in the regression model presented in Table 4 equals that of the corresponding variables in Table 3. In addition, the variables gender and pregnancy were included in the regression model in Table 4. The variable gender was coded 1 for women and 0 for men, while the variable pregnancy was coded 1 for pregnant women and 0 for non-pregnant women and men.

Estimation of the impact of pregnancy on the increased gender differences in sickness absence was based on regression coefficients. We estimated the increased gender differences by including the variables gender, year, and the interaction of gender by year. The regression coefficient of the interaction term provides an estimate of the average annual increase of the gender differences in sickness absence percentage. The impact of pregnancy was estimated to equal the percentage reduction in the value of the interaction term when pregnancy and the annual increase in sickness absence among pregnant women was controlled for, the latter by means of an additional interaction term: pregnancy by year. The estimate was further adjusted for the interaction of pregnancy by age to account for the specific age effect among pregnant women. The percentage of the value of the coefficient of gender by year which remained after control for the annually increased sickness absence among pregnant women and pregnancy by age equaled the percentage of the increased gender difference which applies to non-pregnant women.

Statistical generalization in terms of confidence intervals and significance testing was left out of the following analyses. This is due to characteristics of the data employed, which is the entire Norwegian population rather than a sample thereof.

Pregnant women's sickness absence increased over the period 1993–2007, merely interrupted by short breaks occurring alongside the implementation of a reform in 2004. This change in sickness absence policy instructed the general practitioners to promote the use of graded rather than full time sickness absence in patients who could work part time, unless they had strong medical reasons to do otherwise. It also involved activity requirements for the employee on sickness benefit. This reform was followed by a decrease in total sickness absence of more than 20 percent [41]. It is since then been documented a strong effect of the general practitioners' general preference for graded sickness absence upon the individuals' total long-term sickness absence and risk of exclusion from working life [42]. This effect is present both in men and women, but is not analyzed in pregnant women particularly. However, the reform is unlikely to have caused any bias for the aim of this particular study.

In 1993, the regulations was enjoined by explicit statements that certification of sickness absence should be based on medical grounds – not the employees' social or financial needs. This entailed a short decrease in pregnant women's sickness absence the following year. Until 1998, the employer covered the first 14 days, while the national insurance covered the rest of the remaining period up till 365 days. However, from 1998 onwards, the employers' period was extended with two days, and the period covered by the national insurance was correspondingly shortened. We have accounted for this in the analyses. Until 1999, sickness absence for government employees was not included in the registry, but due to an amendment sickness absence for this group was gradually included in the registry from 2000 and onwards. Due to incomplete registration of state employment, we were unfortunately unable to exclude all state employees from the analyses. Instead, we chose to ignore days of sickness absence compensated to state employees from 2000 and onwards. Although this implies that the total level of sickness absence is slightly underestimated during the entire period, it also ensures that the years prior to 2000 are comparable to those after.

Since 2002, the national insurance has refunded the employers expenses if the sick listed employee is suffering from pregnancy related conditions and if such a refund is applied for. As our estimates based on the public expenditure arising from this scheme suggest that the impact of this is marginal, we have not separated between sickness absence within and without this amendment in the further analyses.

As shown by the chart titles, some of the following graphs and tables show analyses based solely on employees in childbearing age, which means that employees aged 45 years or older were excluded from the analysis. In all regression models, sickness absence percentage is the dependent variable. This implies an interpretation of coefficients where a value of 0.1 indicates that on average the sickness absence increased 0.1 percentage points by one unit increase on the variable.

## Results

The study population includes a total number of 11452 043 annual observations, distributed by 1743 616 unique individuals. Table 1 provides demographic characteristics of the study population at the first and the last year of observation. For simplicity, data for 1993 and 2007 only were included in the table. During the observed period, a growing number of first-time pregnant women were aged 30+ (Table 2).

**Figure 1:**
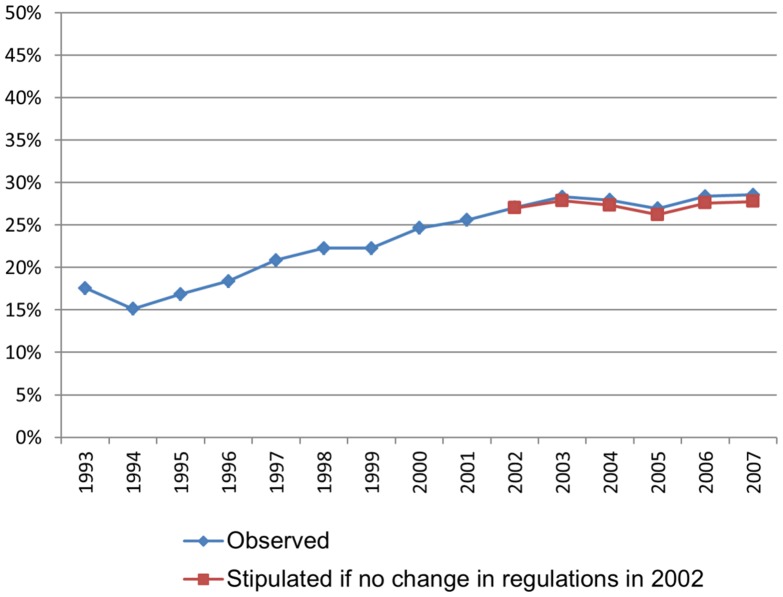
Trend in sickness absence among pregnant women. Sickness absence percent.

The average level of sickness absence among full-time employed pregnant women has increased during the observed period (Figure 1). Refunding of the employers' expenses to pregnant women's sickness absence since 2002 had only a marginal impact on the total average.

**Figure 2:**
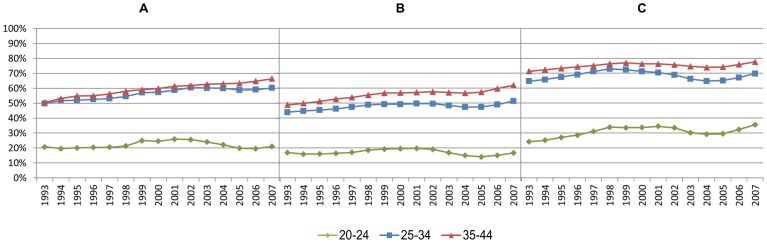
Proportion of the population in fulltime employment. Pregnant women (A), non-pregnant women (B), and men (C).

The proportion of women with full-time income grew rapidly through the period, especially among those aged 35-44 (Figure 2). In the early nineties, the level of full-time employment was much lower among pregnant (A) and non-pregnant women (B), compared to men (C). During the following years, the proportion of full-timers grew particularly rapidly among pregnant women, and at the end of the period, full-time income was even more common among pregnant than non-pregnant women (Figure 2).

**Figure 3:**
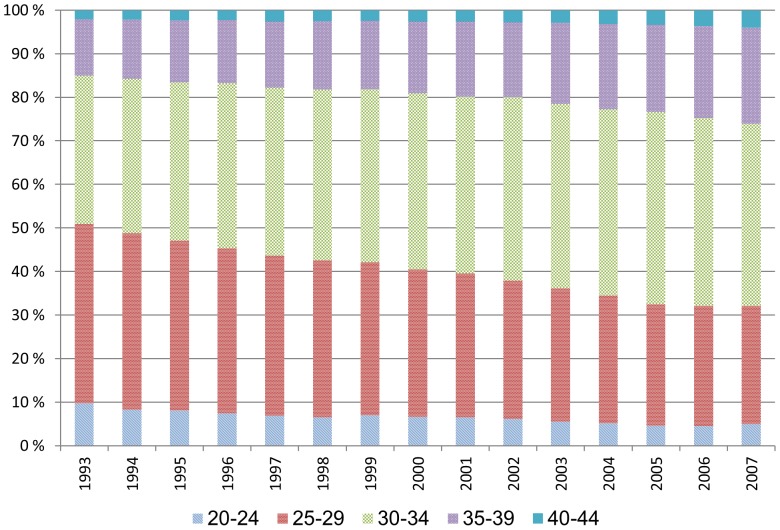
Increased age of pregnant full-time employees.

**Figure 4:**
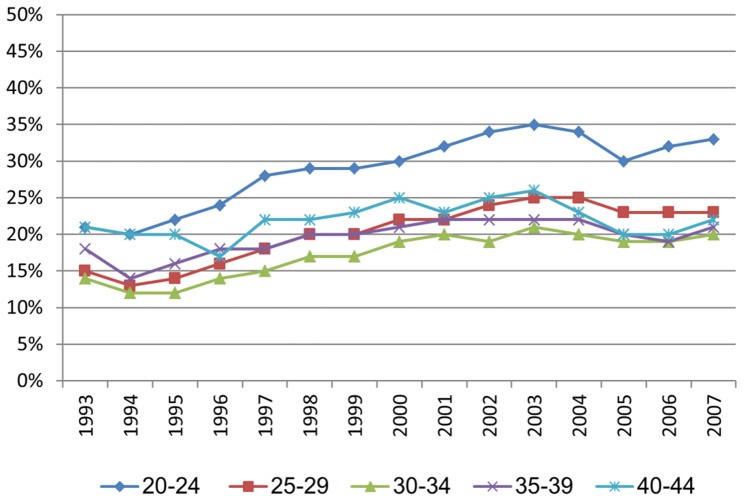
Sickness absence among first-time pregnant women in different age groups.

The average age at first child birth increased steadily and strongly throughout the period (Figure 3). In 1993, both the youngest (20–24) and the oldest (40–44) age group of pregnant women had 21% sickness absence, higher levels than any other age group (Figure 4). Since then, there has been a stronger increase in sickness absence in the youngest age group than in any other age group, whereas the oldest group of pregnant women has only had a weak increase in sickness absence (Table 2).

**Figure 5:**
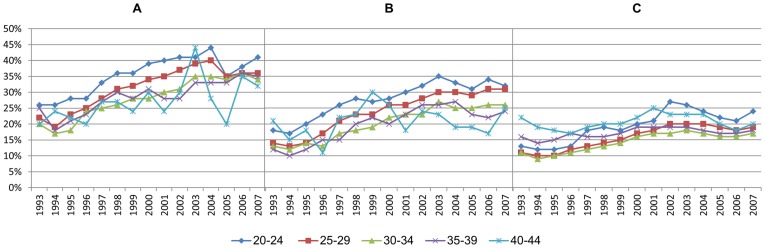
Sickness absence among first-time pregnant women in different age groups. Stratified by educational level: Primary school (A), secondary school (B), and higher education (C).

**Figure 6:**
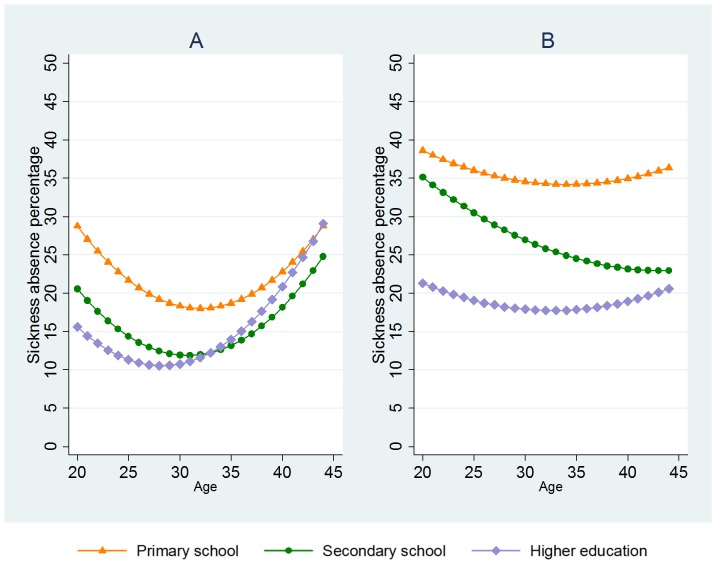
Marginal effects of age and education on sickness absence among first-time pregnant women. Linear regression of the two first and last years of the observed period: 1993–1994 (A), and 2006–2007 (B).

Further stratifying for education (Figure 5), the highest level of sickness absence and the strongest increase was found in younger pregnant women with primary school only (A) or secondary school (B). In these educational groups, the differences between age groups were also the strongest, with stronger increase in younger than older women. In the highest educational group of pregnant women (C), there was less increase in sickness absence, the level of absence was lower, and the there was only little variation between age groups. This interaction between educational level and age for time-trends in sickness absence is presented through regression coefficients in Table 3. The regression is also illustrated in Figure 6, indicating U-shaped associations between age and sickness absence in the start of the observation period (A), whereas at the end of the observation period, there was less of this U-shape (B).

Generally, sickness absence in pregnant women was related to younger (20–24) and older (40–44) age throughout the period, and also lower educational level, (though there were quite few first-time pregnant women aged 40–44 at the start of the observation period). However, the effect of these factors changes over time. Educational level became more defining for sickness absence in pregnant women at the end of the observation period than in the beginning, whereas the effect of age on sickness absence was reduced throughout the period (Figure 6).

According to the multivariate regression (Table 3), pregnant women's sickness absence increased on average 0.64 percent points annually throughout the period (Model 1), which would have been a stronger increase of 0.94 percentage points per year if it was not for increased age at first pregnancy, increased educational level and changes in salary (Model 3).

**Figure 7:**
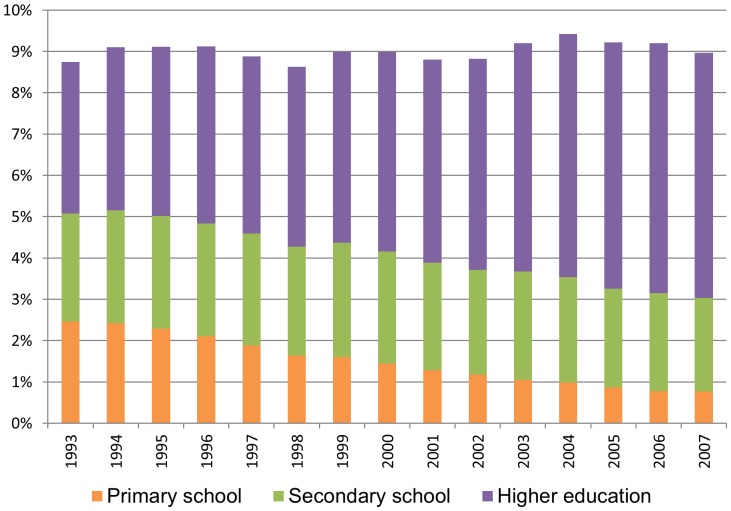
The proportion of full-time employed women aged 20–44 undergoing pregnancy each year is rather stable. The educational level is increasing.

**Figure 8:**
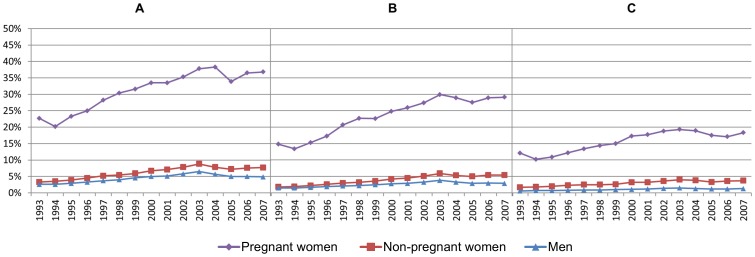
Average percentage of sickness absence among men, pregnant and non-pregnant women. Full-time employees, age 20–44. First-time pregnancies only. Stratified by educational level: Primary school (A), secondary school (B), and higher education (C).

The total proportion of pregnant full-time employees was relatively stable throughout the period, but the educational level within this group increased (Figure 7). The increased sickness absence applied to pregnant women of all educational levels (Figure 8). The overall level of sickness absence was highest among employees with primary school only (A), both for men, pregnant, and non-pregnant women. The overall level of sickness absence was substantially lower among employees with higher education (C).

**Figure 9:**
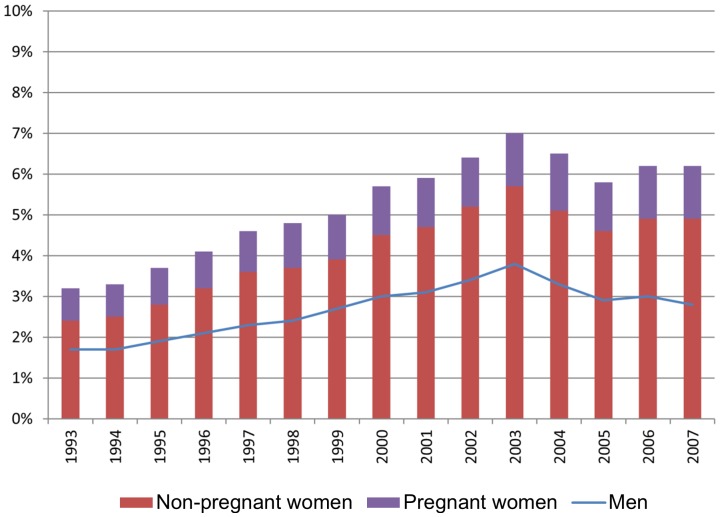
The increased gender differences in sickness absence. 24.98% of the total increase is attributable to sickness absence during pregnancy. Employees in full time employment, age 20–44.

**Figure 10:**
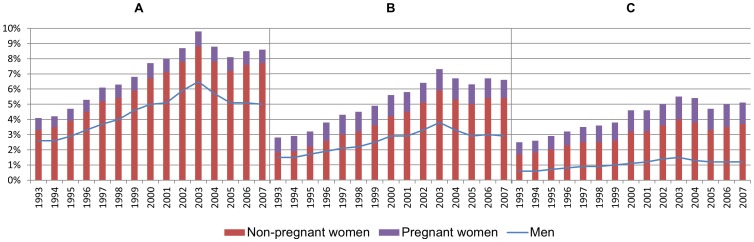
The gender gap in sickness absence among full-time employees. Age 20–44. Stratified by educational level: Primary school (A), secondary school (B), and higher education (C).

The majority of women's increased sickness absence applied to non-pregnant women (Figure 9). The increased gender gap in sickness absence – expressed by the growing distance between the blue line and the top of the columns – applied to all educational levels (Figure 10). This implies that the increased gender gap in sickness absence was not accounted for by pregnant women, regardless of education.

The proportion of the increased gender difference in sickness absence accounted for by absence during pregnancy was estimated applying linear regression models (Table 4). Adjusted for covariates in Model 4, the coefficient of gender indicates that the gender difference in sickness absence increased by 0.15 percentage points each year during the observed period. In Model 5, the value of this coefficient was reduced to 0.11 by control for pregnancy, pregnancy by year, and pregnancy by age. When all decimals were included, controlling for pregnant women's sickness absence led to a 24.98% reduction of the coefficient of gender by year in Model 4. Accordingly, the remaining 75.26% of the increased gender differences applied to non-pregnant women. When heightening the income inclusion criterion to 4 basic units, controlling for pregnancy, pregnancy by year, and pregnancy by age led to a 26.64% reduction of the increased gender differences in sickness absence, when all other control variables were included (results not shown in table).

## Discussion

There was a U-shaped association between age and sickness absence in pregnant women, with considerably more absence (and also far more cases) in the youngest (20–24) than the oldest (40–44) pregnant women. Pregnant women aged 20–24 had the highest rate of sick leave during the entire observational period and also the strongest increase in sickness absence. Consequently, pregnant women's increased sickness absence was not due to higher age at first pregnancy. Sickness absence increased substantially more among pregnant than non-pregnant women, but due to short duration of pregnancy compared to non-pregnancy during employment, pregnancy related absence accounted for no more than 25% of the increased gender difference in sickness absence. These associations are observations of macro-level time trends, and cannot warrant conclusions regarding causality beyond selection effects, i.e. in that postponement of pregnancy would not have increased sickness absence on an individual level. Sickness absence in pregnant women was also related to low educational level. However, the relative effects of age versus educational level changed over time. Educational level became more defining for sickness absence in pregnant women during the observation period, whereas the effect of age on sickness absence was reduced.

Recent investigations differ in their conclusions on whether gender differences in sickness absence in Norway are increasing [5], [6]. Further, pregnancy has previously had a substantial impact on gender differentials in sickness absence in Sweden in the mid 80ies [30]. This study confirms that this was also the case for Norway in the beginning of the 90ies, but also that a majority of the increased sickness absence in the following decade applied to non-pregnant women. Whether a similar development occurred in Sweden during these years remains a question for future research.

Lately higher age among pregnant women has become more common in western countries [15], [16], and in Norway this development has coincided with increased sickness absence among pregnant women. Surprisingly young pregnant women have had the sharpest increase in sickness absence in Norway [7], [8], which is contrary to the prediction that postponement of pregnancies give higher rates of complications and thus also sickness absence. In this paper we have firstly examined if the growing number of pregnant women aged 30 and more still heighten the sickness absence rates through higher overall levels of sickness absence. This is not the case, as younger pregnant women had the highest overall level of sickness absence. Secondly we have examined if pregnant women's increased sickness absence explained the increased gender differentials in sickness absence in Norway from 1993–2007. This is not the case either as most of the increased sickness absence in Norway applied to non-pregnant women. Neither of these questions has previously been addressed.

### Strengths and limitations

The data employed in the analyses have obvious advantages in terms of eliminating the risk for type I and type II error, as well as non-response and self-reporting bias. However, these data also have certain limitations. The register only contains information about sick leave spells covered by the national insurance, leaving spells of shorter duration than 14–16 days out of the register. Special arrangements are made for sickness absence due to certain chronic conditions, in which case the whole spell is covered by national insurance and thereby included in the registry. The previously mentioned amendment from 2002 extended this rule to also apply for pregnancy related sickness absence if this is applied for by the employer, meaning that an additional proportion of pregnancy related sickness absence is included in the registry from 2002 onwards. This represents a potential source of error in terms of overestimating sickness absence during pregnancy after 2002, but our estimates based on the expenditures following from this amendment indicate that the overall impact on the level of sickness absence during pregnancy was small. However, it is not possible to measure whether the impact differs according to the employees' age or education.

Our income based definition of full-time employment also entails some weaknesses. Some full-time working individuals with low income were probably excluded from the analyses, and some part-time employees with high hourly payment were probably included in the analyses. Among these individuals sickness absence will be underestimated. Rising levels of female full-time employment during the observation period probably make the unintentional inclusion of part timers more pronounced in the initial part of the observation period than in the end. If women's sickness absence was somewhat underestimated in the initial part of the observation, this could also have led to an overestimation of the increased gender differences in sickness absence. Income inequalities in Norway are relatively small [43], especially among women [44], which reduces this problem. Still future research could aim at investigate this potential weakness.

Among Norwegian men the income inequalities are larger, but part-time employment is rare; 90% of the employed males had a full-time position in 2002 [45]. This limits the problem of misclassification among men as well.

It is also worth mentioning that a combination of household registers and birth registers were used to identify first time pregnancies, as births were not registered before 1992. This is potentially problematic as women might also live with their partner's children, or the child might live with the father of the child after family dissolution. However, in spite of high levels of gender equality on other areas, children tend to stay with their mother after parental break up in Norway [46], [47], which implies that the risk of misclassification of births is marginal.

Further, women were defined as first time mothers even if they previously had lived with children who were less than 18 years younger than themselves, because these children were regarded as siblings rather than offsprings. This procedure has caused misclassifications in women giving birth younger than 18. However, only 6% of the first-time deliveries in 2004 applied to teenage mothers (including giving birth at 18 and 19), and the frequency of teenage births has decreased substantially since the 70ies [48]. The decreasing number of teenage mothers suggests that the increase in sickness absence among first-time mothers is not due to this misclassification. Further, teenage mothers more often suffer from unemployment and low earnings than older mothers [48], thus some of these misclassified cases are excluded to criterion on full-time income.

### Implications

High and growing levels of sickness absence among pregnant and non-pregnant women entail public expenses and challenges gender equality on the labor market.

In spite of efforts to explain Norwegian women's heightened sickness absence in terms of women's health, pregnancy, double burden of work and family, occupation or norms, the gender differences in sickness absence in Norway largely remains unexplained. Women's heightened sickness absence implies that retrenchment of the sickness benefit will increase the gender pay gap. This backdrop requires cautiousness in policy making.

The sharp increase in sickness absence among pregnant women is striking, especially among the youngest women. Future research should aim at illuminating whether these women somehow are subjected to negative selection. Ongoing initiatives in Norway involve midwives supervising pregnant workers at their workplace. Hopefully this can help reducing sickness absence among pregnant women in a way that meet their needs.

Previous research on health differences, double burden of work and family, labour market segregation and gender specific attitudes have so far not explained the (increasing) gender differences in sickness absence in Norway. Our analyses suggest that the impact of pregnancy on women's sickness absence is still limited, even though sickness absence among pregnant women has increased substantially. The possible impact of growing tension in combining employment and motherhood, medicalization of (pregnancy related) symptoms, or lowered threshold for welfare dependency is not yet established. The relation between gender equality in the society at large and gender differences in sickness absence is a hot topic due to its ambiguity. On the one hand gender differences in sickness absence represent an obstacle to gender equality in the labour marked, on the other hand a generous sickness benefit may be a necessary prerequisite for women in combining work and family building. Future research should beware similar ambiguities in other countries characterized by processes of gender equality enhancing policies. A stepwise, thorough knowledge production is required to ensure that debates about gender inequality are being as evidence based as possible.

## Conclusion

The increase in sickness absence during pregnancy is substantial, but it is not due to higher age among pregnant women. Further, the expansion of the gender gap is mainly due to increasing frequency of sickness absence among non-pregnant women, and about 24.98%of the expansion applies to pregnant women. To conclude, the widening gender gap in sickness absence is not caused by the increasing number of older, pregnant women. The gender gap in sickness absence, the increase in this gap, and the remarkably strong increase in sickness absence in pregnant women (in particular in young women and women with lower education) is generally poorly understood, and needs to be addressed in future studies applying different theoretical viewpoints and methods.
